# Polycomb group genes are required to maintain a binary fate choice in the *Drosophila* eye

**DOI:** 10.1186/s13064-015-0029-7

**Published:** 2015-01-31

**Authors:** Jennifer K Finley, Adam C Miller, Tory G Herman

**Affiliations:** Institute of Molecular Biology, University of Oregon, 1370 Franklin Blvd, Eugene, OR 97403 USA

**Keywords:** Polycomb, PRC1, Photoreceptor, R7, Fate switch, Stochastic, Notch, Senseless/Gfi-1

## Abstract

**Background:**

Identifying the mechanisms by which cells remain irreversibly committed to their fates is a critical step toward understanding and being able to manipulate development and homeostasis. Polycomb group (PcG) proteins are chromatin modifiers that maintain transcriptional silencing, and loss of PcG genes causes widespread derepression of many developmentally important genes. However, because of their broad effects, the degree to which PcG proteins are used at specific fate choice points has not been tested. To understand how fate choices are maintained, we have been analyzing R7 photoreceptor neuron development in the fly eye. R1, R6, and R7 neurons are recruited from a pool of equivalent precursors. In order to adopt the R7 fate, these precursors make three binary choices. They: (1) adopt a neuronal fate, as a consequence of high receptor tyrosine kinase (RTK) activity (they would otherwise become non-neuronal support cells); (2) fail to express Seven-up (Svp), as a consequence of Notch (N) activation (they would otherwise express Svp and become R1/R6 neurons); and (3) fail to express Senseless (Sens), as a parallel consequence of N activation (they would otherwise express Sens and become R8 neurons in the absence of Svp). We were able to remove PcG genes specifically from post-mitotic R1/R6/R7 precursors, allowing us to probe these genes' roles in the three binary fate choices that R1/R6/R7 precursors face when differentiating as R7s.

**Results:**

Here, we show that loss of the PcG genes *Sce*, *Scm*, or *Pc* specifically affects one of the three binary fate choices that R7 precursors must make: mutant R7s derepress Sens and adopt R8 fate characteristics. We find that this fate transformation occurs independently of the PcG genes' canonical role in repressing Hox genes. While N initially establishes Sens repression in R7s, we show that N is not required to keep Sens off, nor do these PcG genes act downstream of N. Instead, the PcG genes act independently of N to maintain Sens repression in R1/R6/R7 precursors that adopt the R7 fate.

**Conclusions:**

We conclude that cells can use PcG genes specifically to maintain a subset of their binary fate choices.

## Background

During development, cells differentiate by making specific sequences of choices among alternative fates. Such choices are typically stable, even when determined by transient events such as the receipt of a signal or the stochastically fluctuating levels of a transcription factor. How is this achieved? Both theory and experimental evidence suggest that the initial commitment to a discrete fate choice depends on regulatory circuits that contain positive feedback [1-6]. Commitment can then be maintained by a combination of two mechanisms: (1) stably expressed sequence-specific transcription factors actively maintain gene expression appropriate to the differentiated cell [1-4,7,8] and (2) transiently expressed sequence-specific transcription factors recruit chromatin modifiers to sites that are then stably marked for transcriptional activation or repression [9,10].

Polycomb group (PcG) proteins are chromatin modifiers that can maintain transcriptional silencing even in the absence of the transcription factors that originally recruited them [11,12]. PcG genes were first identified because of their role in maintaining Hox gene repression along the anterior-posterior axis in animals, and the Hox genes remain the PcG proteins' best characterized targets. However, recent genome-wide analyses in both *Drosophila* and mammals have identified hundreds of other genes that can be bound by one or more PcG complexes in a variety of cell types [13-15]. A disproportionate number of these genes encode transcription factors involved in cell fate specification, leading to the hypothesis that PcG proteins might be used by all cells to maintain multiple aspects of their differentiated or undifferentiated states [14,16]. In support of this hypothesis, loss of PcG genes causes misexpression of many different transcripts and disrupts the fates of multiple cell types. However, whether an individual cell uses PcG proteins to maintain the outcomes of one or more of its successive fate choices during differentiation is difficult to establish given the complexity of the defects caused by broad PcG gene loss.

To understand how cells maintain a long-term commitment to their fate, we have been studying the R7 photoreceptor neurons in the fly eye. An advantage of this system is that we can remove gene function from individual R7 precursors, while leaving most or all surrounding cells unperturbed [17,18]. As a consequence, it is possible to assess the roles of even broadly required genes in R7 fate specification and maintenance. The R1, R6, and R7 neurons are recruited from a pool of equivalent precursor cells and are distinguished from one another by Delta/Notch (Dl/N) signaling [18-22]. The two precursors that adopt the molecularly equivalent R1 and R6 fates are recruited first, fail to receive a Dl signal, and consequently express the R1/R6-specifying transcription factor Seven-up (Svp) [23]. The precursor that normally adopts the R7 fate is recruited second and receives a Dl signal from its new R1 and R6 neighbors. The consequent activation of N within that precursor causes the cell to adopt the R7 fate by: (1) repressing Svp [23-25]; (2) repressing the R8-specifying transcription factor Senseless (Sens) [26,27], which is otherwise stochastically expressed when Svp is absent [25]; and (3) increasing expression of the Sevenless (Sev) receptor [21,22]. How the R7 neurons subsequently maintain their choice of fate is not known.

Here, we show that R7s use the PcG proteins Sex combs extra (Sce) and Sex combs on midleg (Scm) to maintain repression of Sens and prevent a late transformation to an R8-like fate. By contrast, R7s do not require these PcG genes to maintain Svp repression or to prevent late adoption of R1/R6 fate characteristics. We present evidence that the PcG genes act in parallel to N, which initiates Sens repression. We conclude that R7s require PcG genes to maintain just one of the alternative fate choices made during their commitment to the R7 fate.

## Results

### Loss of Sce, Scm, or Pc causes adult R7s to exhibit R8-specific characteristics

**Figure 1:**
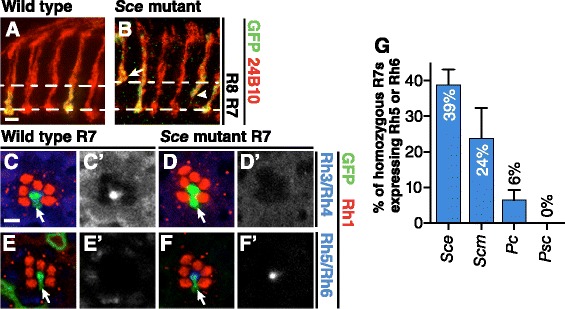
**R7s lacking** ***Sce*** **,** ***Scm*** **, or** ***Pc*** **adopt an R8-like fate. (A,B)** Medullas from adult mosaic animals. Homozygous R7 clones generated by *GMR-FLP*/MARCM express GFP (green). All R axons are labeled with mAb24B10 (red). Scale bar is 5 μm. Wild-type R8 axons terminate in the M3 layer (upper dashed lines) whereas wild-type R7 axons terminate in the M6 layer (lower dashed lines; [28,29]). **(A)** All wild-type (homozygous *FRT82*) R7 axons terminate in M6, the R7 target layer. **(B)** 38.4% ± 9.4% (*n* = 3 brains) of *Sce* mutant R7 axons terminate in M3, the R8 target layer (arrow). A further 7.6% ± 3.4% terminate between the M3 and M6 layers (arrowhead). **(C–F')** Representative adult mosaic ommatidia containing homozygous R7s (green, arrows) generated by *GMR-FLP*/MARCM. Stained with antibodies both against the R1-R6-specific rhodopsin Rh1 (red) and against either the R7 rhodopsins Rh3 and Rh4 (**C–D'**; blue) or the R8 rhodopsins Rh5 and Rh6 (**E–F'**; blue). Scale bar is 5 μm. **(C,C')** Wild-type (*FRT82*) R7s express Rh3 or Rh4 rhodopsins. **(D,D')** Many *Sce* mutant R7s lack Rh3 and Rh4 rhodopsins. **(E,E')** Wild-type (*FRT82*) R7s lack the R8-specific Rh5 and Rh6 rhodopsins. **(F,F')** Many *Sce* mutant R7s express either Rh5 or Rh6 rhodopsin. **(G)** Quantification of R8 rhodopsin expression by homozygous *Sce, Scm*, *Pc*, or *Psc* mutant R7s in adults. Error bars represent SEM. 38.8% ± 4.4% (*n* = 10 retinas) of *Sce*, 23.8% ± 8.5% (*n* = 4 retinas) of *Scm*, and 6.37% ± 3.0% (*n* = 4 retinas) of *Pc* mutant R7s express Rh5 or Rh6. We never observed homozygous wild-type or *Psc* mutant R7s expressing Rh5 or Rh6 and also found that 0/1,090 (*n* = 4 retinas) *Sce/+* and 0/1,150 (*n* = 4 retinas) *Scm/+* R7s expressed Rh5 or Rh6.

To identify genes required for layer-specific targeting of R axons, we performed a genetic screen by creating homozygous mutant R1/R6/R7 precursors within otherwise wild-type animals [17]. We isolated one lethal mutation, *65a*, that causes many R7 axons to terminate in the R8-specific target layer of the optic lobe rather than in the R7-specific target layer [28,29]. We mapped *65a* by meiotic recombination and found that the *65a* chromosome contains a DNA sequence change within the *Sce* coding sequence (predicted to cause a premature stop codon) and that *65a* fails to complement the canonical *Sce*^*1*^ allele for lethality [30]. We confirmed that *Sce*^*1*^ mutant R7 axons also terminate in the R8 target layer (38.4% ± 9.4% (*n* = 3 brains); Figure 1A,B). We conclude that *65a* is an allele of *Sce* and performed all subsequent analyses using the *Sce*^*1*^ allele, as it is considered a null [31].

The phenotype of *Sce* mutant R7s suggested either that Sce is required specifically for proper R7 axon targeting or that Sce prevents R7 neurons from adopting the R8 fate. To distinguish between these possibilities we examined a second R8-specific characteristic, the expression of Rh5 and Rh6 opsins. We found that 39.0% ± 4.4% (*n* = 10 retinas) of *Sce* mutant R7s fail to express either of the R7 opsins, Rh3 and Rh4, (Figure 1C–D') and instead, express the R8 opsins, Rh5 or Rh6 (Figure 1E–G; in nearly all cases the misexpressed R8 opsin is Rh6 and not Rh5). We conclude that Sce is required to prevent R7s from adopting multiple aspects of the R8 fate.

Sce is the fly homolog of the mammalian PcG RING1 proteins and is a core member of Polycomb repressive complex 1 (PRC1) [15,16,31,32]. However, RING1 proteins can also have PRC1-independent activities (reviewed in [33]). To test whether other components of PRC1 might also be required to prevent R7s from adopting the R8 fate, we examined the effect of disrupting the PRC1-associated protein Scm [34-36], and the PRC1 core members, Polycomb (Pc) [15,16,37], and Posterior sexcombs (Psc) [15,38,39]. We found that 23.8% ± 8.5% (*n* = 4 retinas) of R7s homozygous for an *Scm* null allele misexpressed R8-specific opsins, as did 6.37% ± 3.0% (*n* = 4 retinas) of *Pc* null mutant R7s, while *Psc* null mutant R7s did not (Figure 1G). We conclude that multiple PRC1-associated proteins are required to prevent R7s from adopting an R8-like fate.

### Loss of Sce does not cause misexpression of Hox genes in R7s

**Figure 2:**
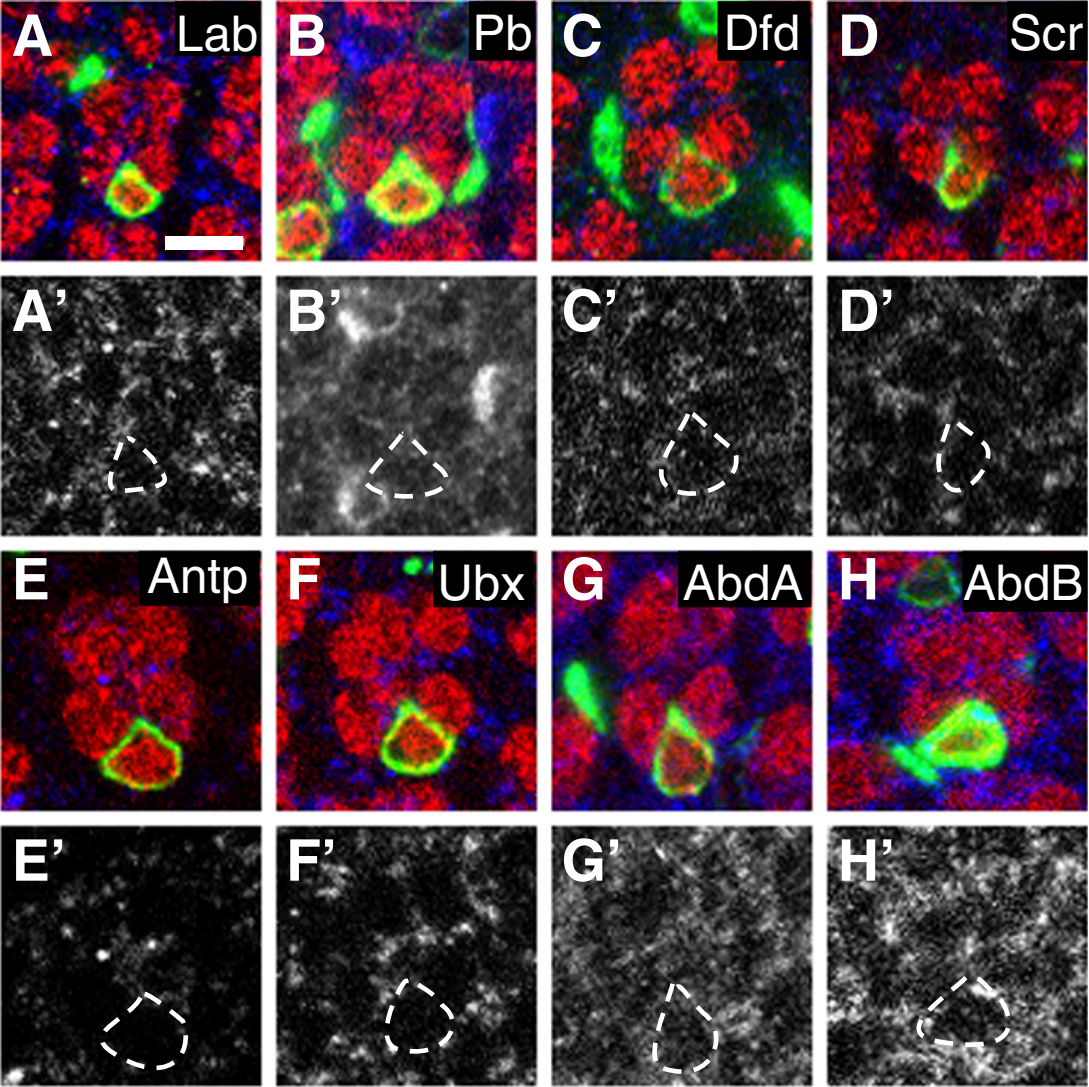
***Sce*** **mutant R7s do not misexpress any of the eight Hox proteins. (A–H')** Representative mosaic ommatidia containing *Sce* mutant R7s (green; dashed outlines) at 24 hr APF, stained with antibodies against: **(A,A')** Labial (Lab); **(B,B')** Proboscipedia (Pb); **(C,C')** Deformed (Dfd); **(D,D')** Sex combs reduced (Scr); **(E,E')** Antennapedia (Antp); **(F,F')** Ultrabithorax (Ubx); **(G,G')** Abdominal-A (AbdA); or **(H,H')** Abdominal-B (AbdB). Scale bar is 5 μm.

PRC1 is required for the long-term transcriptional silencing of specific genes during development. The most common targets of PRC1-mediated silencing in both vertebrates and invertebrates are the Hox genes. While none of the Hox genes is normally expressed in fly photoreceptor neurons, including R8s [40], widespread loss of PcG genes early in fly eye development has been shown to cause ectopic expression of the Hox gene Ultrabithorax [41]. We therefore examined whether loss of *Sce* from R7s might cause them to misexpress any of the eight *Drosophila* Hox genes. We found no detectable Hox protein in *Sce* mutant R7s (Figure 2A-H'), despite observing the expected patterns of Hox expression elsewhere in the same samples. We conclude that the transformation of *Sce* mutant R7s toward the R8 fate is unlikely to be caused by Hox misexpression.

### Sce and Scm are required to maintain the repression of Sens in R7s

**Figure 3:**
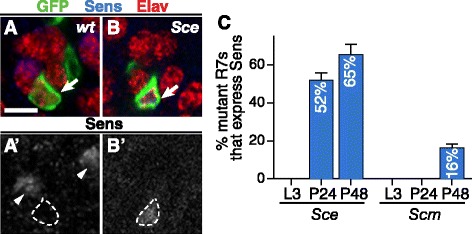
***Sce*** **and** ***Scm*** **are required to maintain repression of Sens in R7s. (A–B')** Representative mosaic ommatidia containing homozygous R7 clones created by *GMR-FLP*/MARCM (green; arrows) at 24 h APF. Scale bar is 5 μm. **(A,A')** Wild-type (homozygous *FRT82*) R7s do not express Sens (dashed outline). Arrowheads point to Sens in R8s. **(B,B')** *Sce* mutant R7 misexpressing Sens (dashed outline). **(C)** Quantification of Sens expression in *Sce* and *Scm* mutant R7s. Error bars represent SEM. No *Sce* mutant R7s express Sens in late larval (L3) eye discs (0/551 R7s in 5 eye discs). 52.1% ± 3.7% (*n* = 10 retinas) of *Sce* mutant R7s express Sens at 24 h APF and 65.5% ± 5.4% (*n* = 7 retinas) do so at 48 h APF. No *Scm* mutant R7s express Sens at L3 (0/447 R7s in 3 eye discs) or 24 h APF (0/108 R7s in 3 retinas). 16.3% ± 1.9% (*n* = 5 retinas) of *Scm* mutant R7s express Sens at 48 h APF.

The R8 fate is normally specified by the transcription factor Sens [26,27], which directly regulates transcription of both R8-specific rhodopsins [27] and the R8-specific axon targeting molecule Capricious [42,43]. Ectopic Sens expression in R7s causes them to adopt R8-like characteristics [25,27,43]. We therefore examined whether the loss of PRC1 components from R7s might cause derepression of Sens. Alternatively, the R7-specific transcription factor Prospero (Pros) is required to prevent R7s from misexpressing R8 rhodopsins [44] and forming synaptic boutons in the R8 target layer [25], suggesting that loss of PRC1 components might instead cause loss of Pros. R7s are first specified during the late third larval stage (L3) of development and subsequently select synaptic targets and express opsins during the pupal stage. We found that no *Sce* mutant R7s express Sens in L3 animals but that 52.1% ± 3.7% (*n* = 10 retinas) of *Sce* mutant R7s misexpress Sens at 24 h after puparium formation (h APF) and 65.5% ± 5.4% (*n* = 7 retinas) do so at 48 h APF (Figure 3A–C). By contrast, 99.1% ± 2.6% (*n* = 10 retinas) of *Sce* mutant R7s continue to express Pros. We conclude that Sce is required to maintain Sens repression in R7s but is not required to maintain Pros expression. Similarly, we found that, while no *Scm* mutant R7s express Sens at L3 (0/447 R7s in 3 eye discs) or 24 h APF (0/108 R7s in 3 retinas), 16.3% ± 1.9% (*n* = 5 retinas) of *Scm* mutant R7s express Sens at 48 h APF (Figure 3C). We conclude that Sce and Scm are required to maintain Sens repression in R7s, which otherwise begin to misexpress Sens.

### N is not required to maintain repression of Sens in R7s

**Figure 4:**
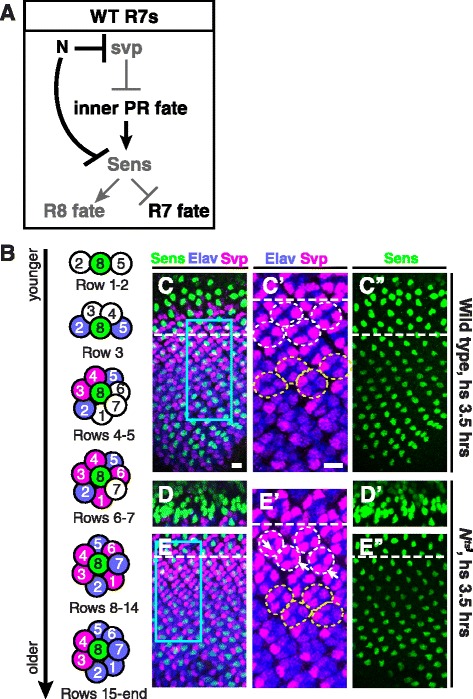
**N is not required to maintain repression of Sens in R7s. (A)** Model of Sens repression in R7s [25]. N is activated in R7 precursors in row 6, repressing Svp and directing R7 precursors toward either of two inner photoreceptor fates, R7 or R8. N also represses Sens in R7 precursors, causing them to become R7s. **(B)** Wild-type ommatidial development. R neurons express Elav (blue); R1, R3, R4, and R6 express Svp (magenta); and R8 expresses Sens (green). **(C–C'')** A wild-type larval eye disc after upshift to 31°C for 3.5 h. The dashed line in C–C'' lies along row 6. The box in C corresponds to panel C'. **(C)** Svp is expressed in R3 and R4 from row 4 onward and in R1 and R6 beginning in row 6. **(C')** Ommatidia in rows 6-10 contain four Svp-positive cells. White dashed circles indicate ommatidia in rows 7-8, and yellow dashed circles ommatidia in rows 10-11. **(C'')** Sens is expressed in R8s only. **(D–E'')** A *N* ^*ts1*^ mutant larval eye disc after upshift to the non-permissive temperature, 31°C, for 3.5 h. D–E'' are images of the same eye disc. **(D,D')** Sens is expressed in extra cells within rows 0–3, consistent with N's role in specifying R8s [46]. **(E,E')** Svp is expressed as in wild-type until row 7. In rows 7–8 Svp is misexpressed in R7s (white dashed circles; arrows indicate ommatidia with Svp-positive R7s), since these R7s were in rows 5–6 during the temperature shift and never experienced N activation. However, from row 9 onward, Svp is never derepressed in R7s (e.g. yellow dashed circles in rows 10–11). **(E'')** From row 4 onward, Sens is never expressed in R7s, despite inactivation of N and lack of Svp. Scale bars are 10 μm.

We next wanted to determine the relationship between Sce and Scm and the previously defined gene regulatory pathway that controls R7 fate. R1/R6s and R7s are recruited from a pool of equivalent precursors and are specified as photoreceptor neurons by high levels of receptor tyrosine kinase (RTK) signaling [21,22,45]. A second pathway, N, distinguishes the R1/R6 and R7 fates: precursors in which N is not activated express Svp and consequently become R1/R6s; those in which N is activated fail to express Svp and therefore become R7s [21,22,45]. During normal development, the first two precursors to be recruited by RTK signaling occupy the so-called “R1/R6 niche” in which they are protected from N activation and therefore become R1/R6s; the third precursor to be recruited occupies the “R7 niche” and is consequently exposed to Dl, causing activation of N and adoption of the R7 fate. We previously showed that N represses Sens in R1/R6/R7 precursors lacking Svp, thereby preventing them from adopting the R8 fate ([25]; Figure 4A). We therefore wanted to examine the relationship between Sce and Scm and N in the regulation of Sens.

We first wanted to determine whether, like Sce and Scm, N is required to maintain Sens repression. Alternatively, N might be required specifically to initiate Sens repression during R7 specification and, unlike Sce and Scm, be dispensable for its later maintenance. To distinguish between these possibilities, we used a temperature-sensitive *N* allele, *N*^*ts1*^, to remove N function from R7 cells after their initial specification. We raised *N*^*ts1*^ mutant animals at the permissive temperature until the late L3 stage and then upshifted them to the non-permissive temperature for 3.5 h [47,48]. Because a single L3 eye disc contains a gradient of ommatidia of different ages [45], this single upshift allowed us to examine the effects of removing N from R7s at multiple stages of their development (Figure 4B). Each row of ommatidia is 1.5 h older than the row immediately anterior; each R7 we examined had therefore been approximately two rows younger when it lost N activity.

To confirm that our temperature upshift effectively removed N activity, we first examined R7s that had not received a Dl signal prior to upshift. R7 precursors are normally recruited and exposed to Dl in rows 5–6, express the N-dependent reporter *mdelta0.5-lacZ* by rows 7–8, and fail to express Svp [18]. We found that, while wild-type R7s exposed to the non-permissive temperature never expressed Svp (Figure 4C,C'), 52% of the *N*^*ts1*^ mutant R7s that were in rows 5 and 6 at the initial time of upshift misexpressed Svp (Figure 4E,E' (arrows)). We conclude that this approach quickly and substantially eliminates N from R7s.

We next examined Sens expression. R1/R6/R7 precursors that lack N during fate specification express Svp, which represses Sens [25]; we would therefore not expect to observe Sens expression by *N*^*ts1*^ mutant R7s that were in rows 5 and 6 at the time of upshift. However, we found that loss of N from older R7s does not cause them to misexpress Svp (Figure 4E,E', yellow dashed circles indicate ommatidia in rows 10 and 11), allowing us to assess whether N is later required to maintain Sens repression. We found that *N*^*ts1*^ mutant R7s that were in rows 7 or higher at the time of upshift never expressed Sens (Figure 4E,E''), despite their lack of Svp. We conclude that, unlike Sce and Scm, activated N is not required to maintain repression of Sens. This result suggests that Sce and Scm do not require activated N in order to maintain Sens repression and are therefore likely to act in parallel to N. However, N might yet act upstream of Sce and Scm if instead the initial pulse of N activation during R7 fate specification is sufficient to positively regulate them. We therefore next wanted to test whether Sce and Scm can repress Sens independently of N in R1/R6/R7 precursors.

### *Sce* and *Scm* are dispensible for Sens repression in R1/R6s but are required to maintain Sens repression in R7s that are generated in the absence of N

**Figure 5:**
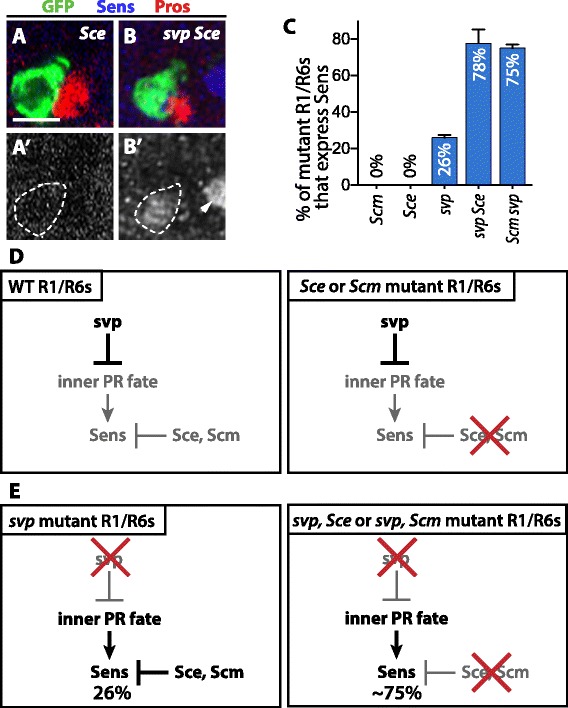
***Sce*** **and** ***Scm*** **are required to maintain Sens repression in the absence of Svp and N** ***.*** **(A–B')** Representative 48 h APF mosaic ommatidia in which the cells occupying the R1/R6 niche are homozygous for a particular chromosome arm (green), stained with antibodies against Sens (blue). In each ommatidium shown, the cell occupying the R7 niche is wild-type and expresses the R7-specific marker Prospero (Pros; [44]; red). Scale bar is 5 μm. **(A,A')** Neither *Sce* nor *Scm* mutant R1/R6s, which express Svp, misexpress Sens (dashed outline; quantified in **(C)**). **(B,B')** Loss of *Sce* or *Scm* from *svp* mutant R1/R6s significantly increases the proportion that misexpress Sens (dashed outline; quantified in **(C)**). The arrowhead in B' indicates Sens within an R8 from an adjacent ommatidium. **(C)** Quantification of mutant R1/R6s that express Sens at 48 hr APF. Error bars represent SEM. No *Sce* or *Scm* mutant R1/R6s express Sens (*n* = 318 in 5 retinas and *n* = 353 in 4 retinas, respectively). 25.9% ± 1.4% (*n* = 6 retinas) of *svp* mutant R1/R6s express Sens. Loss of *Sce* or *Scm* from *svp* mutant R1/R6s greatly increases the proportion that express Sens (77.6% ± 7.5% (*n* = 4 retinas) and 75.0% ± 1.9% (*n* = 7 retinas), respectively). **(D,E)** Model for the regulation of Sens by *Sce* and *Scm* in R1/R6 precursors. **(D)** Wild-type R1/R6s normally express Svp, which prevents expression of Sens. This Sens repression does not require *Sce* or *Scm*, since loss of *Sce* or *Scm* from otherwise wild-type R1/R6s does not result in Sens derepression. **(E)** R1/R6s lacking Svp stochastically adopt one of the two “inner” photoreceptor fates, R8 or R7, depending on whether they express Sens. Those that do not initially express Sens require *Sce* and *Scm* to maintain repression of Sens.

To test whether Sce and Scm repress Sens independently of N, we examined the effect of removing *Sce* or *Scm* from R1/R6/R7 precursors that occupy the R1/R6 niche and consequently do not contain activated N. In these cells, Sens repression is normally established by Svp [25]. We found that *Sce* and *Scm* mutant R1s and R6s never expressed Sens during larval or pupal development (Figure 5A,A',C), nor did they express R8-specific rhodopsins in adult. We conclude that Sce and Scm are not required to maintain the Sens repression that is established by Svp (Figure 5D).

**Figure 6:**
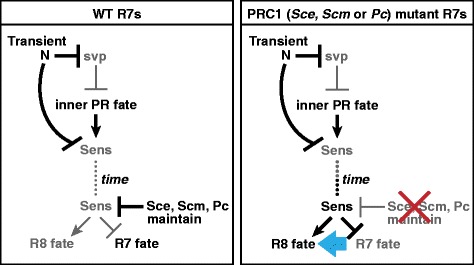
**Model for the regulation of Sens by Sce, Scm, and Pc in R7s.** N is transiently activated in precursors that occupy the R7 niche. As a consequence, these precursors do not express Svp and will adopt one of the “inner” photoreceptor fates, R8 or R7. Transient N also represses Sens, preventing the precursors from becoming R8s and causing them instead to become R7s. These R7s remain prone to expressing Sens stochastically and, independently of N, use Sce, Scm, and Pc to ensure that Sens remains permanently off.

However, cells that adopt the R7 fate do not express Svp. To test whether Sce and Scm repress Sens in R7s independently of N, we therefore next wanted to examine the effect of removing *Sce* or *Scm* from R1/R6/R7 precursors that lack both Svp and N. We previously found that *svp* mutant precursors in the R1/R6 niche stochastically express Sens and become R8s or keep Sens repressed and become R7s despite their lack of N activation [25]. This gave us an opportunity to test whether Sce and Scm repress Sens in precursors that adopt the R7 fate independently of both Svp and N by examining the effect of removing *Sce* or *Scm* from *svp* mutant precursors that occupy the R1/R6 niche. We compared the proportions of *svp* single and *svp Sce* or *Scm svp* double mutant R1/R6 precursors that express Sens. We found that while 26% of *svp* single mutant precursors stochastically express Sens in mid-pupae, a significantly greater proportion of *svp Sce* (78%) and *Scm svp* (75%) double mutant R1/R6s did so (Figure 5B–C), indicating that loss of Sce or Scm causes derepression of Sens in these cells. We conclude that Sce and Scm are required to maintain the repression of Sens that is initiated stochastically in the absence of Svp and N (Figure 5E). Sce and Scm therefore act independently of—that is, in parallel with—N to maintain Sens repression (Figure 6).

## Discussion

### Loss of *Sce* or *Scm* specifically affects the choice between the R7 and R8 fates

Early loss of PcG genes causes widespread and complex fate transformations [41,49]. The *GMR-FLP*/MARCM system allowed us to remove *Sce* and *Scm* function specifically from post-mitotic R1/R6/R7 precursors, allowing us to probe these genes' roles in the limited number of binary fate choices that R1/R6/R7 precursors face. In order to adopt the R7 fate, these precursors must choose to: (1) become neurons in response to high RTK activity—they would otherwise become non-neuronal cells; (2) fail to express Svp in response to N activity—they would otherwise become R1/R6s; and (3) fail to express Sens in response to N activity—they would otherwise become R8s. We found that loss of *Sce* or *Scm* from R7s specifically compromises maintenance of the last of these choices. By contrast, we found no evidence that PcG genes maintain either of the other two choices. We examined *Sce* mutant R7s throughout larval and pupal development and found none that misexpressed Svp, nor did we observe *Sce* or *Scm* mutant R7s that displayed other R1/R6 characteristics, such as large rhabdomeres positioned at the periphery of the ommatidium or expression of the R1-R6-specific rhodopsin Rh1; [28,29,45]). While loss of the Abelson kinase was recently shown to cause R neurons to lose expression of the neuronal marker Elav and switch to a non-neuronal pigment cell fate [50], we found that *Sce* and *Scm* mutant R1/R6s and R7s maintain expression of Elav and the photoreceptor-specific protein Chaoptin (for example, Figure 1B), indicating that their commitment to a neuronal fate is also independent of PcG gene function. We conclude that R7s use Sce and Scm to maintain repression of one but not all alternative binary fate choices.

### By what mechanism(s) might Sce and Scm be repressing Sens?

The Sens-encoding region is bound by Pc in *Drosophila* embryos and by Sce in *Drosophila* larvae [51-53], suggesting that Sens is directly regulated by these proteins in at least some cell types. However, because of the technical difficulty in isolating sufficient quantities of chromatin specifically from R7 cells, we were unable to determine whether PcG proteins bind the Sens locus in R7s. It remains possible, therefore, that Sce, Scm, and Pc maintain Sens repression indirectly in R7s—however, our evidence suggests that they do so independently of their canonical role in repressing Hox genes.

We observed considerable differences in the strengths of the R7 defects caused by loss of *Sce*, *Scm*, *Pc*, or *Psc*. One possibility is that these proteins do not contribute equally to PRC1's gene-silencing ability. Indeed, the fly genome contains a second *Psc*-related gene that plays a redundant role with *Psc* in some cells, possibly accounting for the lack of defect in *Psc* mutant R7s [38,39]. Alternatively, the different wild-type PcG proteins may perdure to different degrees within the mutant R7 clones (the cells that divide to generate the mutant R1/R6/R7 precursors contain a wild-type copy of the mutant gene). We attempted but were unable to measure the time course of Sce and Scm protein levels in *Sce* and *Scm* mutant R7s, respectively, to test their perdurance directly. However, we think perdurance is likely, as we have found that Gal80 perdures until early pupal development within *GMR-FLP*/MARCM-induced R7 clones [16].

### Regulation of Sce and Scm in R1/R6s and R7s

We found that Sce and Scm are required to maintain Sens repression in R7s generated either in the presence or absence of N activity (Figure 6). What might be regulating the deployment of Sce and Scm in these cells? One possibility is that Sce and Scm repress Sens in R1/R6/R7 precursors by default, since these cells never normally express Sens. However, we found that neither Sce nor Scm is required to maintain the repression of Sens that is established by Svp. Alternatively, Sce and Scm may be deployed to repress Sens as part of a cell's initial commitment to the R7 fate. As mentioned above, wild-type Sce or Scm protein is likely to perdure in newly created homozygous *Sce* or *Scm* mutant R7s, respectively, leaving open the possibility that these genes are required not only for the maintenance but also for the establishment of the R7 fate. Previous work showed that the NF-YC subunit of the heterotrimeric transcription factor nuclear factor Y (NF-Y) is also required to maintain Sens repression in R7s [43]. Like the PcG proteins [32], NF-YC is broadly expressed in all photoreceptor neurons [43] and is not sufficient to cause R7s to adopt R8 fates, indicating that NF-YC is not responsible for the specific role of PcG proteins in R7s. However, the resemblance between the R7 defects caused by loss of Sce, Scm, and NF-YC suggests that NF-Y may participate in PRC1 function. In support of this possibility, loss of the NF-YA subunit from *Caenorhabditis elegans* also causes defects similar to those caused by loss of the PcG gene *sop-2*, including derepression of the Hox gene *egl-5* [54].

## Conclusions

PcG proteins have been shown to silence many regulators of development in addition to their canonical Hox targets, suggesting that PcG proteins are likely to play broad roles in maintaining cell fate commitments [16,52]. However, whether PcG proteins are used to maintain specific binary fate choices as cells differentiate is unclear. In fact, the opposite is true during stem cell differentiation, when the repression of terminal differentiation genes by PcG proteins must instead be relieved [55,56]. In this paper, we have identified a role for PRC1-associated PcG proteins in maintaining a specific binary fate choice made during adoption of the R7 fate—a choice that does not involve Hox gene regulation or misregulation. We found that the same PRC1-associated proteins are not required to maintain two other binary fate choices that R7s must make. We conclude that PcG genes are indeed used to maintain some though not all binary fate choices.

## Methods

*65a* was induced with ethyl methanesulfonate by standard methods [57]. Other mutations used were: the null alleles *Sce*^*1*^ (for all *Sce* mutant data presented; [30,31]), *svp*^*e22*^ [23], *Scm*^*D1*^ [30,58], *Pc*^*XT109*^ [59], *Psc*^*e24*^ [60], and the temperature-sensitive hypomorph *N*^*ts1*^ [47]. Homozygous wild-type or mutant R1/R6/R7 precursors were created by *GMR-FLP*-induced mitotic recombination between *FRT*-containing chromosomes [17]. Homozygous cells were labeled by the MARCM technique [61] with either *act-Gal4 UAS-Synaptotagmin (Syt)-GFP* (axon terminals) or *act-Gal4 UAS-mCD8-GFP* (cell bodies). As described previously [18], homozygous mutant cells do not begin to express green fluorescent protein (GFP) until approximately 12 h APF; homozygous *Sce* and *Scm* mutant R7s are therefore unmarked in larval eye discs but constitute approximately 11% of the R7s present [18].

Tissues were dissected, fixed, and stained as described previously [25]. Confocal images were collected on a Leica SP2 microscope and analyzed with Leica, Fiji (http://fiji.sc/Fiji; [62]) or SoftWoRx v.2.5 (Applied Precision, Issaquah, WA) software.

We obtained mouse anti-Chaoptin (24B10; 1:200), mouse anti-Elav (9F8A9; 1:10), rat anti-Elav (7E8A10; 1:5), mouse anti-AbdB (1A2E9; 1:100), mouse anti-Scr (6H4; 1:10), and mouse anti-Antp (4C3 and 8C11; 1:100) from the Developmental Studies Hybridoma Bank; rabbit anti-Rh1 (1:1,000) from D. Ready (Purdue University); mouse anti-Rh3 (1:10), anti-Rh4 (1:10), anti-Rh5 (1:10), and anti-Rh6 (1:50) from S. Britt (UCHSC, Denver); rabbit anti-Rh6 (1:1,000) from C. Desplan (New York University); guinea pig anti-Sens (1:500) from H. Bellen (Baylor College of Medicine); mouse anti-Pros (mR1A, 1:1,000) from C. Doe (University of Oregon); mouse anti-Svp (1:500) from Y. Hiromi via C. Doe (University of Oregon); chicken anti-GFP (1:500) from Abcam (Cambridge, MA); guinea pig anti-Dfd (1:100) from W. McGinnis (University of California, San Diego); mouse anti-AbdA (1:400) from D. Duncan (Washington University, St Louis); rabbit anti-Pb (1:50) from T. Kaufman (Indiana University); mouse anti-Ubx (1:20) from R. White (University of Cambridge); rabbit anti-Lab (1:100) from F. Hirth and H. Reichert (University of Basel); and rabbit anti-GFP (1:1,000), phalloidin conjugated to Alexa Fluor 555 (1:10), and all secondary antibodies (goat IgG coupled to Alexa Fluor 488, 555 or 633 (1:250)) from Molecular Probes (Eugene, OR).

In addition, we used biotinylated secondary antibodies and fluorophore-conjugated streptavidin (donkey anti-rabbit IgG Biotin-SP (1:200), donkey anti-mouse IgG Biotin-SP, donkey anti-guinea pig IgG Biotin-SP, streptavidin-conjugated Cy3 (1:500), and streptavidin-conjugated Alexa Fluor 633 (1:500) from Jackson Immuno Research (West Grove, PA)) to enhance detection of Hox proteins but still observed no expression in *Sce* mutant R7s (data not shown).

## Abbreviations

Dl: Delta
GFP: Green fluorescent protein
h APF: Hours after puparium formation
L3: Third Larval stage
N: Notch
NF-Y: Nuclear factor Y
Pc: Polycomb
PcG: Polycomb group
PRC1: Polycomb repressive complex 1
Pros: Prospero
Psc: Posterior sex combs
RTK: Receptor tyrosine kinase
Sens: Senseless
Sce: Sex combs extra
Scm: Sex combs on midleg
Svp: Seven-up
Syt: Synaptotagmin
